# Myocardial deformation after radiotherapy: a layer-specific and territorial longitudinal strain analysis in a cohort of left-sided breast cancer patients (BACCARAT study)

**DOI:** 10.1186/s13014-020-01635-y

**Published:** 2020-08-20

**Authors:** Valentin Walker, Olivier Lairez, Olivier Fondard, Gaëlle Jimenez, Jérémy Camilleri, Loïc Panh, David Broggio, Marie-Odile Bernier, Dominique Laurier, Jean Ferrières, Sophie Jacob

**Affiliations:** 1 Pôle Santé-Environnement (PSE-SANTE), Service de recherche sur les effets biologiques et sanitaires des rayonnements ionisants (SESANE), Laboratoire d’épidémiologie des rayonnements ionisants (LEPID), Institute for Radiological Protection and Nuclear Safety (IRSN), BP17, 92262 Fontenay-aux-Roses cedex, France; 2 Department of Cardiology, Rangueil University Hospital, 31059 Toulouse, France; 3 Cardiac Imaging Centre, Rangueil University Hospital, 31059 Toulouse, France; 4 Medical School of Rangueil, University Paul Sabatier, 31400 Toulouse, France; 5 Department of Cardiology, Clinique Pasteur, 31300 Toulouse, France; 6 Department of Radiation Oncology (Oncorad), Clinique Pasteur, 31300 Toulouse, France; 7 Department of Cardiac Arrhythmia, Clinique Pasteur, 31300 Toulouse, France; 8 Department of dosimetry, Institute for Radiological Protection and Nuclear Safety (IRSN), Fontenay-aux-Roses, France; 9 Division of Health and Environment, Institute for Radiological Protection and Nuclear Safety (IRSN), Fontenay-aux-Roses, France; 10 Medical School of Purpan, University Paul Sabatier, 31000 Toulouse, France; 11 INSERM, UMR1027, 31000 Toulouse, France

**Keywords:** Radiation therapy, Cardiac toxicity, Echocardiography, Multilayer strain, Coronary arteries

## Abstract

**Background:**

Radiotherapy for breast cancer (BC) and its resulting cardiac exposure are associated with subclinical left ventricular dysfunction characterized by early decrease of global longitudinal strain (LS) measurement based on 2D speckle-tracking echocardiography. Recent software allows multi-layer and segmental analysis of strain, which may be of interest to quantify and locate the impact of cardiac exposure on myocardial function and potentially increase the early detection of radiation-induced cardiotoxicity. The aim of the study was to evaluate whether decrease in LS 6 months after radiotherapy is layer-specific and if it varies according to the left ventricular regional level and the coronary arterial territories.

**Methods:**

LS was measured at baseline before radiotherapy and 6 months post-radiotherapy. The LS was obtained for each myocardial layer (endocardial, mid-myocardial, epicardial), left ventricular regional level (basal, mid, apical) and coronary artery territory (left anterior descending artery (LAD), circumflex artery, right coronary artery).

**Results:**

The study included 64 left-sided BC patients. Mean age was 58 years, mean doses to the heart, the left ventricle and the LAD were respectively 3.0, 6.7 and 16.4 Gy. The absolute decrease of LS was significant for the three layers (endocardial: − 20.0 ± 3.2% to − 18.8 ± 3.8%; mid-myocardial: − 16.0 ± 2.7% to − 15.0 ± 3.1%; epicardial: − 12.3 ± 2.5% to − 11.4 ± 2.8%, all *p* = 0.02), but only the relative decrease of LS in the endocardial layer was close to be significant (− 4.7%, *p* = 0.05). More precisely, the LS of the endocardial layer was significantly decreased for the most exposed parts of the left ventricle corresponding to the apical level (− 26.3 ± 6.0% vs. -24.2 ± 7.1%, *p* = 0.03) and LAD territory (− 22.8 ± 4.0% vs. -21.4 ± 4.8%, *p* = 0.03).

**Conclusion:**

Six months post-radiotherapy, LS decreased predominantly in the endocardial layer of the most exposed part of the left ventricle. For precise evaluation of radiotherapy-induced cardiotoxicity and early left ventricular dysfunction, the endocardial layer-based LS might be the most sensitive parameter.

**Trial registration:**

ClinicalTrials.gov: NCT02605512, Registered 6 November 2015 - Retrospectively registered.

## Background

Radiotherapy (RT) is a major component of breast cancer treatment. Despite its benefits, it is now commonly accepted that breast cancer RT can be associated with long-term cardiac complications, including coronary artery diseases, due to the presence of cardiac tissues in the irradiation field [1–3]. Long before the onset of clinically detectable cardiac events, sensitive parameters of left ventricular myocardial dysfunction based on echocardiography can be investigated. Two-dimensional speckle-tracking echocardiography is a semi-automated quantitative technique for assessment of strain, a measure of myocardial deformation to evaluate the myocardial systolic function. Global longitudinal strain (LS) is often considered as an optimal parameter of deformation for the early detection of sub-clinical left ventricular dysfunction [4, 5]. Many studies on early myocardial dysfunction after BC RT showed a significant decrease in global LS among left-sided breast cancer patients at different times post-RT, from few weeks to 3 years [6–14], whereas no measurable alteration of left ventricular ejection fraction (LVEF) was observed. Moreover, some previous works showed an association between the global LS decrease after BC RT and the mean heart dose or the mean left ventricular dose [15].

However, the left ventricular wall of the heart is composed of three myocardial layers: endocardial, mid-myocardial, and epicardial. Of these 3 layers, the endocardial layer is the most susceptible of ischemic injury [16] and potentially radiotherapy-induced subclinical ischemic injury. Recent softwares allow multi-layer strain analysis [17, 18], but separate evaluation of endocardial, mid-myocardial and epicardial myocardial deformation has never been analyzed in breast cancer patients treated with RT. A careful evaluation of these layers, in particular the endocardial layer, might increase the early detection of radiation-induced cardiotoxicity in this context.

Cardiac exposure due to breast cancer RT is not homogeneous [19]. Highest cardiac radiation doses are likely to be delivered to the anterior part of the heart and the left ventricle, including the left anterior descending coronary artery (LAD), and are observed in the apex and in the apical-anterior segment where some hot spots >50Gy can be found [20]. One study based on strain imaging detected a correlation between the reduction in regional myocardial function (basal level, mid-level and apical level) and the local radiation dose [10] as the decrease in global LS at the apical level was the most important. Heterogeneity of doses among coronary arteries was also demonstrated [19, 21], but it was never considered for LS analysis whereas left ventricular segmentations provide segmental strains that can be assigned to coronary arterial territories [22]. Thus, analysis of territorial myocardial function (LAD, circumflex artery (Cx) and right coronary artery (RCA)) might also be relevant in the context of early detection of RT-induced cardiotoxicity.

Based on the BACCARAT prospective cohort of left and right BC patients treated with 3D-CRT, we had presented a 6-month interim follow-up analysis on a secondary outcome measure defined by a decrease of global LS from baseline to 6 months after RT [15]. In the continuation of these previous results, the aim of this new paper was to evaluate among left-sided BC patients whether this decrease in global LS at the scale of the left ventricle was myocardial layer-specific, depending on regional level and coronary arterial territories, and whether coronary arteries doses were associated with territorial strain changes.

## Patients and methods

### Study population

**Fig. 1:**
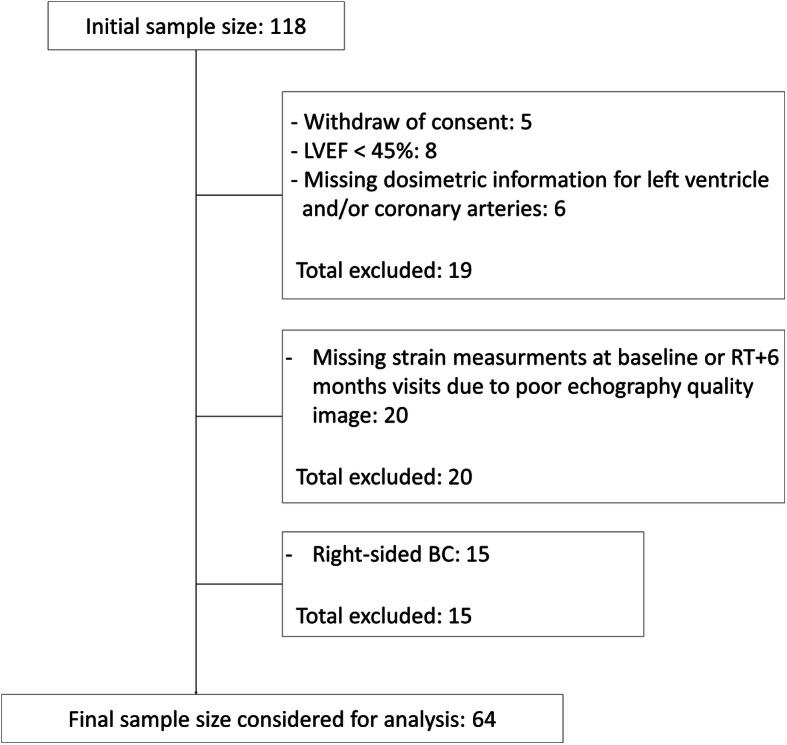
CONSORT-like diagram for the multilayer strain analysis cohort obtained from the BACCARAT population

The BACCARAT study initially included 118 female patients of the Clinic Pasteur Toulouse from October 2015 to December 2017, aged 40 to 75 years old, mainly with left unilateral BC, and in a smaller proportion with right-sided unilateral BC. All patients were treated with adjuvant 3D-CRT after breast conserving surgery or mastectomy, without chemotherapy. Five patients withdrew consent, 8 patients had abnormal LVEF before RT (LVEF < 45%) and 6 patients without available cardiac dosimetry were excluded. We excluded patients with echocardiographies for which the image quality was too low for a reliable assessment of longitudinal strain (*n* = 20) remaining 79 patients [15, 23]. For the analysis presented here, we focused on left-sided BC patients, and finally, the patient study group consisted of 64 patients (Fig. 1). With a follow-up of 6-months after RT, none of the 64 patients included had undergone chemotherapy.

### Radiotherapy treatment and radiation doses evaluation

All patients were treated with 3D-CRT. The prescription dose was delivered over 5 weeks: either 50 Gy in 25 daily fractions of 2 Gy or 47 Gy in 20 daily fractions of 2.35 Gy. The methods to evaluate radiation doses in BACCARAT patients were presented elsewhere [15, 19, 23]. Dose-Volume-Histogram (DVH) for the heart was generated by the Clinic Pasteur radiotherapy department. Before RT, a coronary computed tomography angiography (CCTA) was performed for all patients as planned in the BACCARAT protocol. For dosimetric evaluation of coronary arteries, the simulation CT scan, the CCTA, the RT dose and RT structure files in DICOM format were used. Merging anatomical information from the simulation CT scan and the CCTA was performed. Once inserted in ISOgray TPS (version 4.2, Dosisoft, Cachan, France; http://www.dosisoft.com/en/radiotherapy/planning-products.html), manual delineation was performed for the left ventricle (LV), the left anterior descending artery (LAD), the left circumflex artery (Cx) and the right coronary artery (RCA). Using the 3D dose matrix generated during treatment planning and the new delineated substructures, DVH for LV and coronary arteries were generated with ISOgray TPS by the dosimetric department of IRSN in collaboration with the Clinic Pasteur radiotherapy department. We thus obtained mean doses for the following cardiac structures: whole heart, left ventricle, left anterior descending artery, circumflex artery and right coronary artery.

### Transthoracic echocardiography

**Fig. 2:**
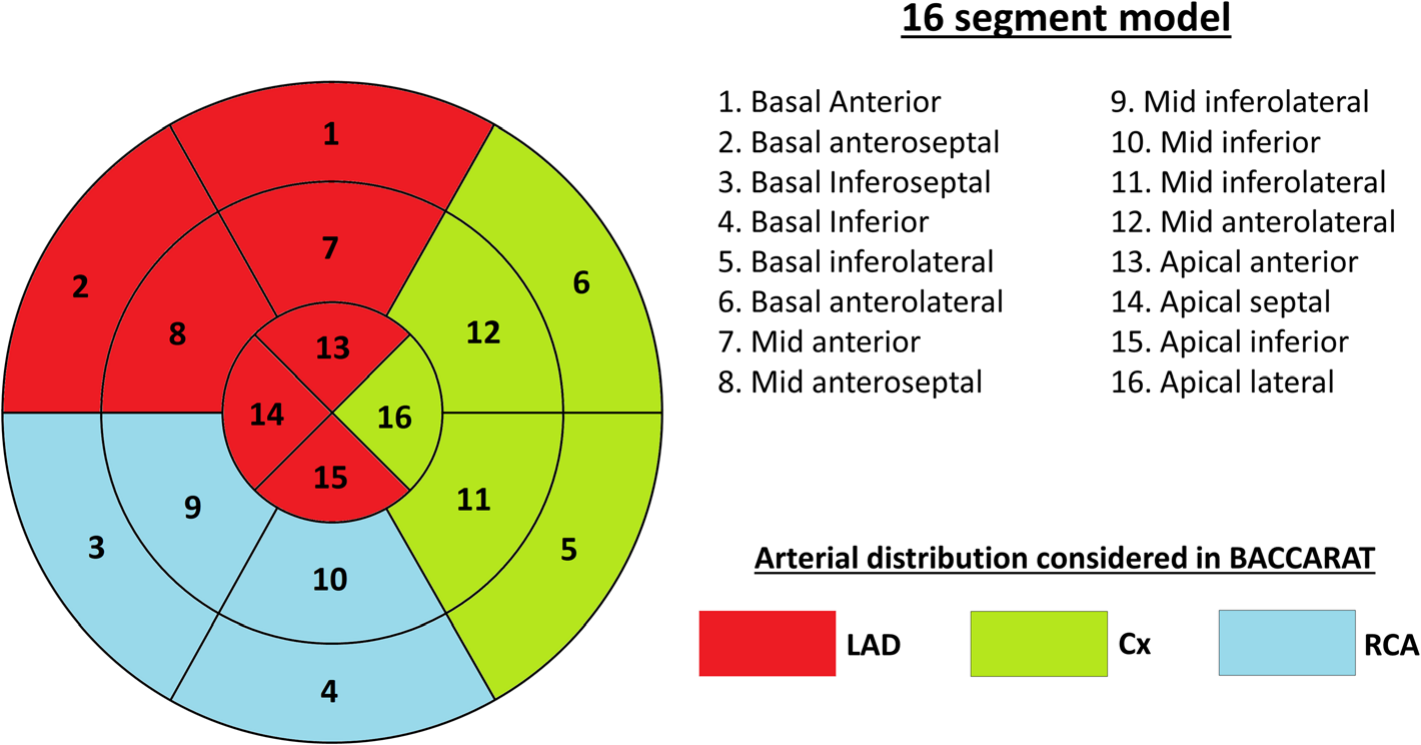
“Bull-eye” presentation of the left ventricle: 16-segmental model and coronary artery territories. Legend: LAD - Left Anterior Descending artery; Cx - Circumflex artery; RCA - Right Coronary Artery

Transthoracic echocardiography was performed at baseline before RT and 6 months after RT with ultrasound Acuson S2000 device (Siemens Medical Solutions USA, Inc. Malvern, USA), using a 3 MHz transducer. Image analysis was independently performed by a single blinded observer unaware of clinical data. Longitudinal strain (LS) measurement was evaluated using two-dimensional speckle tracking [24] using a 16-segment model as recommended by the American Society of Echocardiography guidelines [25]. Global LS for the whole left ventricle was obtained for each myocardial layer (endocardial, mid-myocardial and epicardial). Mean LS for each regional level (basal, mid and apical) and for each coronary artery territory (Territorial Longitudinal Strain (TLS) LAD, TLS Cx and TLS RCA) [22] was calculated as the mean of segmental LS included in these levels (Fig. 2). In particular, the specific LS corresponding to the segments of the LAD (TLS LAD), was based on the average of segments 1, 2, 7, 8, 13, 14 and 15 as indicated in Fig. 2.

### Statistical analysis

Continuous variables are presented with mean and standard deviation or median and interquartile range values. Categorical values are presented with percentages. Student’s *t*-test or Wilcoxon non-parametric test was used to compare continuous variables, adapted to paired samples for the comparison of echocardiographic variables before RT and 6 months after RT. Comparison of layer-specific LS at baseline and RT + 6 months were performed, mean relative change was evaluated (Mean = V6 – V0 / V0). Specific analysis of segmental strains values according to regional level or coronary arteries territorial areas was performed, and the evolution of LS in these levels from baseline to RT + 6 months was analyzed. We compared these evolutions according to the group of exposure (“High” for patients with cardiac doses >66th percentile of dose distribution, “Low” for others). Given the exploratory nature of this work, we presented unadjusted *p*-values for comparisons, but in order to take into account multiple testing in these comparisons we also applied the Holm–Bonferroni method, a step-down procedure performed after conducting the multiple comparison tests. Finally, *p*-value < 0.05 was considered statistically significant. All statistical analysis was performed using SAS statistical software for Windows (Version 9.4 TS1M4 – SAS Institute, Cary, NC).

## Results

### Study population

Sixty-four left-sided breast cancer patients were included in the analysis. Baseline characteristics of the population are shown in Table 1. The mean age at inclusion was 58 ± 9 years. Concerning cardiac risk factors, 14 (22%), 20 (31%) and 28 (44%) patients had hypertension, hypercholesterolemia and a BMI over 25 kg/m^2^, respectively. Moreover, 8% of patients had diabetes, and 47% were current smokers. The mean dose received by the left ventricle was more than twice as high as the mean dose received by the whole heart (6.68 ± 3.36 versus 3.05 ± 1.31, *P* = < 0.0001). For coronary arteries exposure, the highest mean dose was found in the LAD (16.41 ± 7.41 Gy), while the lowest mean dose was found in the RCA (0.71 ± 0.37 Gy).

**Table 1:** Baseline characteristics of the study population

	Left-sided BC patients ***n*** = 64
**Age** in years, mean ± SD	58 ± 9
**Type of cancer**, n (%)	
In situ	11 (17%)
Invasive	53 (83%)
**Surgery**, n (%)	
Breast conserving	61 (95%)
Mastectomy	3 (54%)
**Regional lymph node irradiation**	22 (34%)
*Supraclavicular alone*	*1*
*Internal mammary alone*	*2*
*Both*	*19*
**Body mass index** in kg/m^2^, mean ± SD	24.5 ± 4.2
**Smoking**, n (%)	
Never-smokers	34 (53%)
Former smokers	20 (31%)
Current smokers	10 (16%)
**Systolic blood pressure,** in mmHg, mean ± SD	119 ± 12
**Diastolic blood pressure** in mmHg, mean ± SD	75 ± 10
**Hypertension**, n (%)	14 (22%)
**Diabetes**, n (%)	5 (8%)
**Hypercholesterolemia**, n (%)	20 (31%)
**Cardiac doses** in Gy	
**Whole Heart**	
Mean ± SD	3.05 ± 1.31
Min – Max	0.87–6.37
**Left Ventricle**	
Mean ± SD	6.68 ± 3.36
Min – Max	1.16–13.42
**Left Descending Artery**	
Mean ± SD	16.41 ± 7.41
Min – Max	1.68–34.63
**Circumflex Artery**	
Mean ± SD	1.65 ± 0.82
Min – Max	0.53–4.34
**Right Coronary Artery**	
Mean ± SD	0.71 ± 0.37
Min – Max	0.14–2.50

*BC* Breast Cancer, *SD* Standard Deviation

### Multilayer evolution of the global longitudinal strain

Echocardiography parameters are displayed in Table 2. Left ventricular ejection fraction (LVEF) remained within normal range after RT. A significant decrease in global longitudinal strain was observed for each myocardial layer, but the highest mean relative change from baseline to RT + 6 months was observed in the endocardial layer (− 4.7%, *p* = 0.05) whereas for other layers, the mean relative change was slightly lower.

**Table 2:** Comparison of baseline and follow-up measurements of echocardiographic data

	Baseline ***n*** = 64	6 months after RT ***n*** = 64	***P***-value	Relative change (%)***p***-value
**LVEF**, %	61 ± 7	60 ± 9	0.073	Na.
**GLS, %**				
Endocardial layer	−20.0 ± 3.2	−18.8 ± 3.8	**0.02**	−4.7%; **0.05**
Mid-myocardial layer	−16.0 ± 2.7	−15.0 ± 3.1	**0.02**	− 4.4%; 0.11
Epicardial layer	−12.3 ± 2.5	−11.4 ± 2.8	**0.02**	−4.2%; 0.25

*LVEF* Left Ventricular Ejection Fraction, *GLS* Global Longitudinal Strain, *Na.* Not assessed

### Left ventricular regional evolution of the longitudinal strain in the endocardial layer

**Fig. 3:**
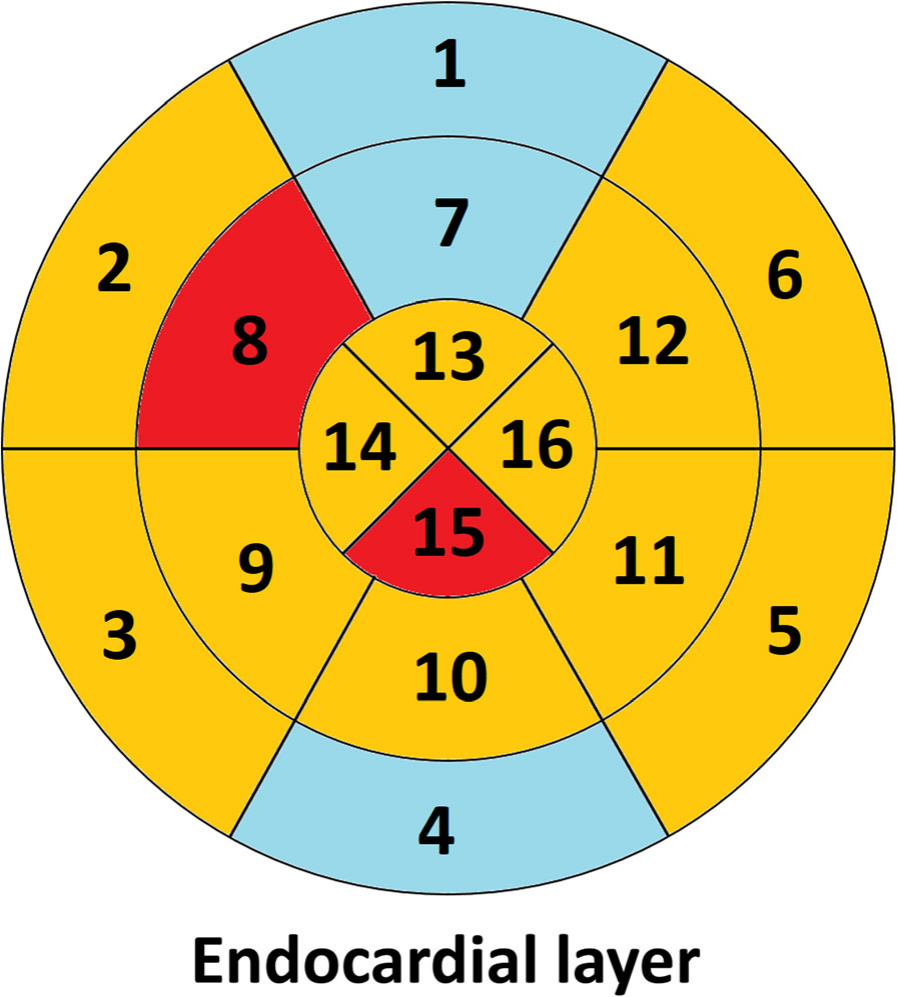
Segmental analysis of the endocardial layer according to longitudinal strain based on bull’s eye representation (16 segment model). Legend. Red for segments with significant decrease in longitudinal strain from baseline to RT + 6 months (but not significant after Holm-Bonferroni method for multiple testing on 16 tests); Orange for segments with non-significant decrease in longitudinal strain; Blue for segments with non-significant increase in longitudinal strain

Among the three regional left ventricular levels, a significant decrease of the LS was observed only in the apical level (− 26.3 ± 6.0% vs. -24.2 ± 7.1%, *p* = 0.03). After Holm-Bonferroni method for multiple testing for 3 tests, this decrease did not reach statistical significance. While separating patients into two groups according to their exposure to the left ventricle **(**Table 3**)**, the regional analysis showed that LS decreased significantly after RT at the apical level in the highly exposed group corresponding to the 22 patients with LV Dose >66th percentile = 8.6 Gy (− 25.5 ± 6.3 at V0 to − 22.7 ± 6.9 at V6; *p* = 0.04), which did not remain significant after multiple testing correction. More precisely, the segmental analysis of strain values **(**Fig. 3**)** showed a decrease in all segments of the apical level, with significant deteriorations in the apical inferior segment (segment 15) and the mid-anteroseptal segment (segment 8), but not significant after Holm-Bonferroni method.

**Table 3:** Regional analysis of longitudinal strains in the endocardial layer

		Low dose to the LV ***n*** = 42		High dose to the LV ***n*** = 22	
		V0	V6	V0	V6
**Basal level, %**	**Mean ± SD**	−16.7 ± 5.6	−17.1 ± 5.0	−19.0 ± 3.5	−17.8 ± 3.2
	***p*-value**	**0.52**		**0.10**	
**Mid-level, %**	**Mean ± SD**	− 18.6 ± 3.0	−17.0 ± 5.2	−17.6 ± 3.4	−17.5 ± 4.7
	***p*-value**	**0.06**		**0.90**	
**Apical level, %**	**Mean ± SD**	−26.7 ± 5.8	−25.0 ± 7.2	−25.5 ± 6.3	−22.7 ± 6.9
	***p*-value**	**0.18**		**0.04**^**a**^	

*LV* Left Ventricle. Low dose to the LV corresponds to patients receiving < 8.6 Gy to the LV (66th percentile of dose distribution among the 64 patients). High dose to the LV corresponds to patients receiving > 8.6 Gy. ^a^Not significant after Holm-Bonferroni method for multiple testing

### Coronary arteries territorial evolution of the endocardial layer longitudinal strain

The coronary arteries territorial analysis showed no significant decrease for the Cx and the RCA. However, an alteration of the LS was observed for the LAD territory (− 22.8 ± 4.0% vs. -21.4 ± 4.8%, *p* = 0.03). While separating patients into two groups according to their exposure to the left ventricle (Table 4), an alteration of the LS was observed for the LAD territory in the highly exposed group corresponding to patients receiving > 8.6Gy to the LV (− 22.7 ± 3.4 at V0 to − 20.7 ± 4.5 at V6; *p* = 0.05).

**Table 4:** Territorial analysis of longitudinal strains in the endocardial layer for the coronary arteries

		Low dose to the LV ***N*** = 42		High dose to the LV ***N*** = 22	
		V0	V6	V0	V6
**TLS - LAD, %**	**Mean ± SD**	− 22.9 ± 4,3	−21.8 ± 5,0	− 22.7 ± 3.4	−20.7 ± 4.5
	***p*-value**	**0.20**		**0.05**^**a**^	
**TLS - Cx, %**	**Mean ± SD**	− 19.0 ± 4.9	− 17.5 ± 5.1	−19.5 ± 3.8	−17.9 ± 5.5
	***p*-value**	**0.10**		**0.25**	
**TLS - RCA, %**	**Mean ± SD**	− 16.2 ± 4.7	−16.2 ± 5.2	−16.9 ± 4.8	−15.6 ± 5.1
	***p*-value**	**0.97**		**0.35**	

Left Ventricle; *TLS* Territorial Longitudinal Strain, *LAD* Left Anterior Descending artery, *Cx* Circumflex artery, *RCA* Right Coronary Artery. Low dose to the LV corresponds to patients receiving < 8.6 Gy to the LV (66th percentile of dose distribution among the 64 patients). High dose to the LV corresponds to patients receiving > 8.6 Gy. ^a^Not significant after Holm-Bonferroni method for multiple testing

**Fig. 4:**
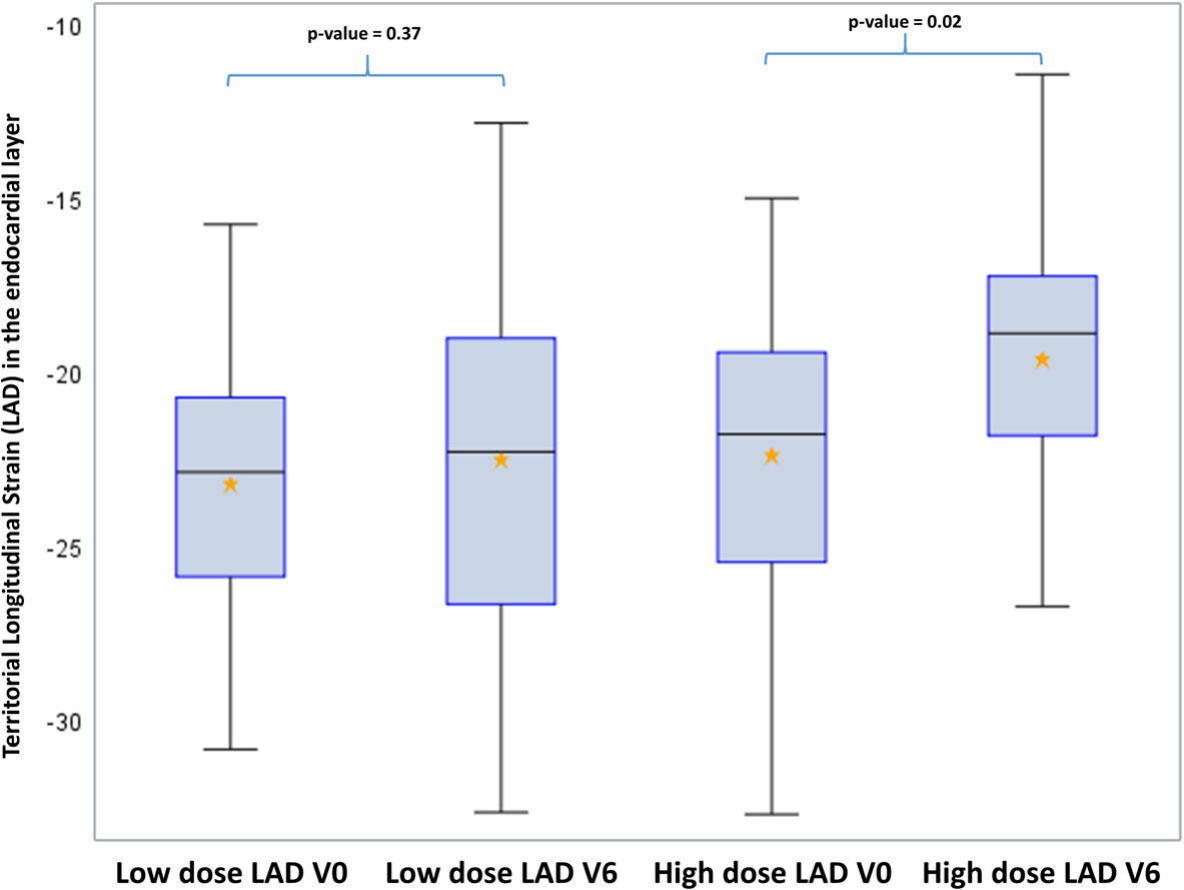
Longitudinal strain in the endocardial layer for the LAD territory according to the exposure level of the LAD. Legend: LAD = Left Anterior Descending artery; High dose for patients with LAD dose > 19.9 Gy; Low doses for patients with LAD dose < 19.9 Gy). V0 = Baseline; V6 = 6 months after radiotherapy. NB: After Holm-Bonferroni method for multiple testing (2 tests), the *p*-value of 0.02 remain significant

**Fig. 5:**
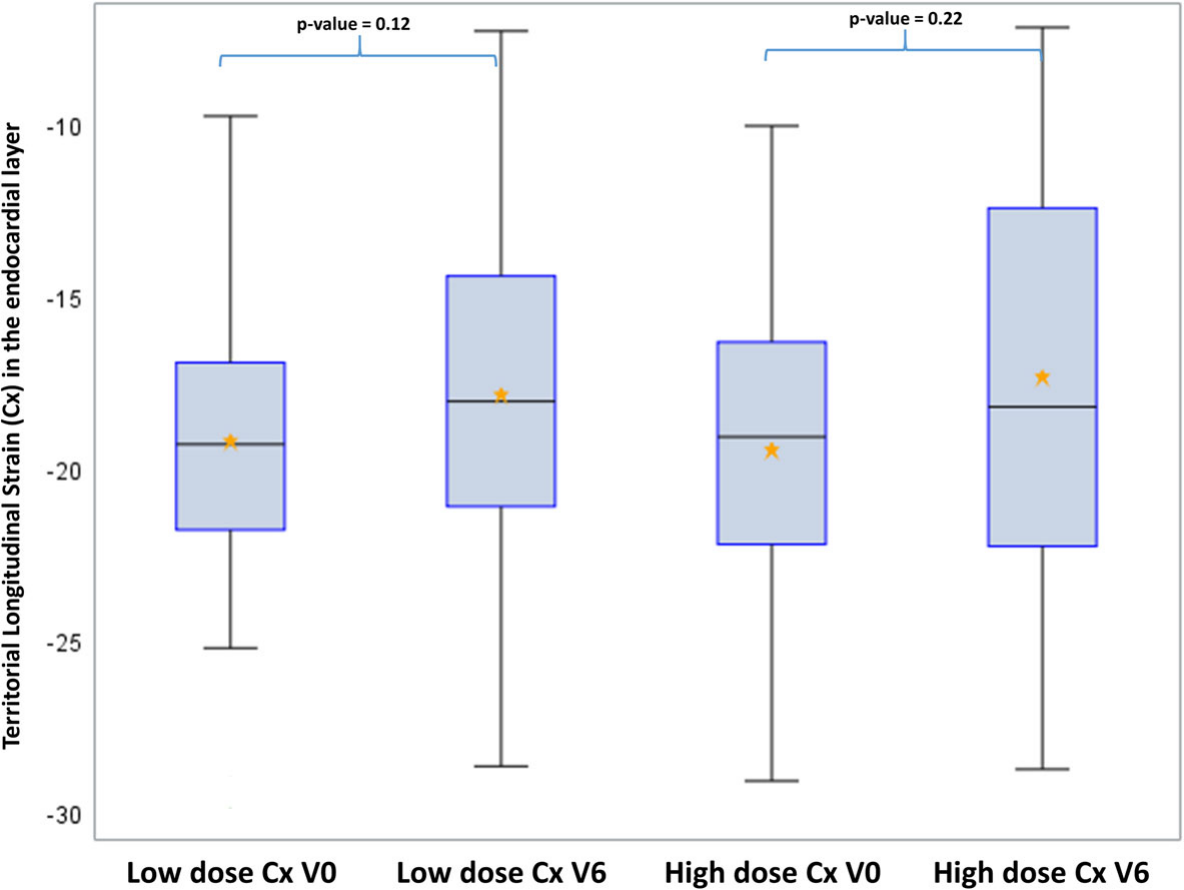
Longitudinal strain in the endocardial layer for the Cx territory according to the exposure level of the Cx. Legend: Cx = Circumflex artery; High dose for patients with LAD dose > 1.8 Gy; Low doses for patients with LAD dose < 1.8Gy). V0 = Baseline; V6 = 6 months after radiotherapy

**Fig. 6:**
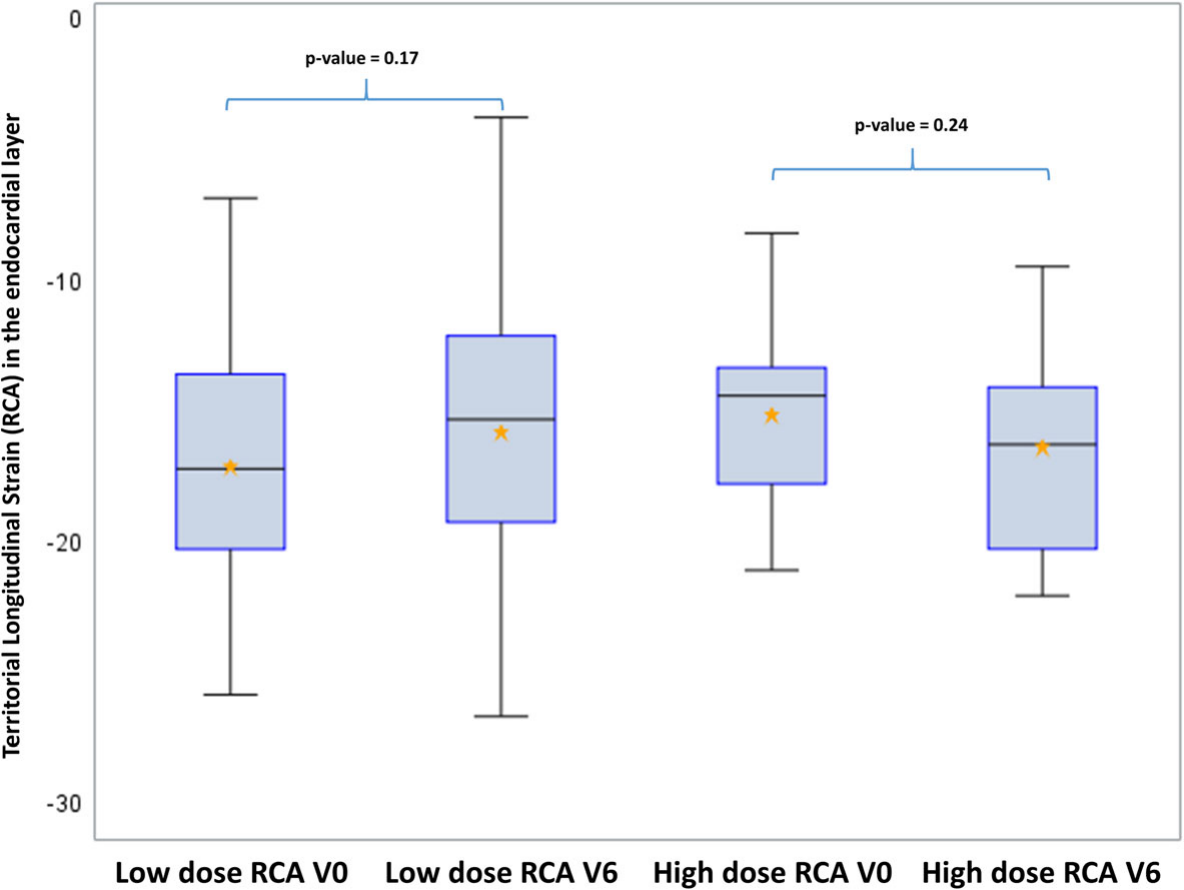
Longitudinal strain in the endocardial layer for the RCA territory according to the exposure level of the RCA. Legend: RCA = Right Coronary Artery; High dose for patients with LAD dose > 0.8 Gy; Low doses for patients with LAD dose < 0.8Gy). V0 = Baseline; V6 = 6 months after radiotherapy

Moreover, longitudinal strain decrease in the LAD territory could be associated with the dose level of the LAD with a significant decrease observed in the highly exposed group corresponding to the 22 patients with LAD Dose >66th percentile = 19.9 Gy (*p* = 0.02), and this result remained significant after Holm-Bonferroni method for 2-tests comparisons. No significant difference could be observed in less exposed patients, corresponding to a decrease of LS from − 22.3% at V0 to − 19.5% at V6 (Fig. 4). In comparison, no significant difference could be observed for the Cx and the RCA at the highest exposures, even by taking into account the precise dose to these substructures (Figs. 5 and 6).

## Discussion

At the scale of the whole left ventricle, the previously reported deterioration of LS 6 months after BC RT [15], is confirmed on the three layers of the myocardial wall. However, our study suggests that the LS strain change may be more relevant in the endocardial layer, in particular in the most exposed areas of the left ventricle, corresponding to the apical region and the LAD territory.

### Difference of strain according to myocardial layers

The LS difference according to the myocardial layers that we observed in either baseline or RT + 6 months values, with higher values in the endocardial layer and lower values in the epicardial layer, has been previously observed [26]. In normal heart, contraction is greater in the endocardial layer than in the epicardial layer [27] and the difference in amplitude of myocardial contraction between the endocardial and epicardial regions is related to the orientation pattern of myocardial fiber in the heart as the subendocardium is predominantly composed of longitudinal myocardial fiber. Moreover, the longitudinal left ventricular mechanics are predominantly governed by the subendocardial layer of the myocardium, which may explain the significant decrease in global LS from baseline to RT + 6 months visits in the three layers. However, with greater contraction and higher energy requirements, endocardial layer is more susceptible to injury which may explain that the relative decrease in LS was slightly higher in the endocardial layer.

### Location of LS deterioration

Unlike chemotherapy, which impact on myocardial function can be considered global at the scale of the left ventricle, RT affects the heart in a more localized way as the apical level of the left ventricle is particularly exposed to the tangential beams of the RT [19]. This may explain the strongest decrease of the LS at the apical level as previously observed [6]. Moreover, in a previous study of patients with left-sided breast cancer [12], the segments with a significant strain reduction just after RT and 3 years post-RT were similar to those found in our study, particularly with regards to the mid-anterolateral segment and the apical-inferior segment. However, the association between LS decrease and cardiac dose is far to be clear and our correlations between LS decrease and doses to the different cardiac structures were low (under 0.3), as previously observed in several other studies [6, 10, 11].

Concerning the coronary arteries territorial analysis of LS, we found significant deterioration of the LS for the LAD. The strongest impact on the LAD territory could be explained by the fact that the segments related to this coronary artery received highest radiation doses [19]. Moreover, patients with the highest LAD doses (> 20 Gy) were those with a clear LS deterioration as illustrated in Fig. 3.

### LS of the endocardial layer and LAD territory

It is commonly accepted that the endocardium is the most susceptible target to ischemic injury [28, 29]. Moreover, it has been shown that LS of the endocardial layer was superior to other layers to identify significant coronary artery disease [16]. On the other hand, previous researches pointed toward a relationship between the location of coronary stenosis and radiation dose after RT, primarily at the LAD [30–33]. Our results indicated that the impact of RT on LS could be observed particularly in the endocardial layer within the LAD territory, which is concordant with these previous publications. LAD is known to be the most affected coronary artery with long term follow up after BC RT. Therefore, although LS is not a direct marker of coronary artery disease, a significant deterioration of endocardial layer LS in the segments of the LAD territory with a short follow-up could be a potential marker of other cardiac disease such as an early marker of potential more severe injury in the LAD.

### Limitations

Several limitations should be mentioned. The first point is not specific of our study, but a general limitation of global LS to know how the changes in LS might translate into clinical cardiac outcome parameters (morbidity/mortality). In the context of chemotherapy, it has been showed that there is a correlation and a predictive value of GLS decrease on the later presence of the outcome CTRCD (Cancer Therapeutics-Related Cardiac Dysfunction) defined by a decrease in LVEF of at least 10% to a value < 53% [4]. However, such CTRCD may be reversible in some cases and do not translate into clinical cardiac morbidity. Although the decrease in longitudinal strain and LVEF appears to at least partially persist throughout the treatment it is unknown what their evolution will be in subsequent years, and whether early deformation measurements will predict persistent decreases in LVEF or symptomatic heart failure. In the context of general population, a wide Danish study established that lower GLS was associated with a higher risk of a composite endpoint defined by incident heart failure, acute myocardial infarction or cardiovascular death (HR 1.12 [1.08–1.17], *p* < 0.001 per 1% decrease) [34]. In the context of radiotherapy, it is still unknown whether changes in LS will translate into clinical cardiac morbidity or mortality. In summary, based on different studies in different contexts, use of GLS measurement in the specific context of radiotherapy-treated patients is more and more frequent as an additional parameter (in particular to LVEF) to potentially predict later cardiac morbi/mortality, but it has still to be investigated and validated in observational studies with long follow-up. As a second limitation, our study is a prospective study but based on short follow-up and a relatively small population of left-sided BC patients, which did not provide information on the value of specific myocardial deformation parameters in the prognosis of cardiac complication, in particular injury to the LAD. Further studies with clear clinical endpoints will be required to determine the clinical significance of our findings. In particular, the observation of decreased LS in the LAD territory as well as in the apical region of the left ventricle should be compared with observations from computed tomography coronary angiography analysis [19, 31]. Furthermore, multi-layer strain analysis is controversial [35], limited by poor reproducibility and important variability [26]. Our results were exploratory and should be confirmed by other studies.

## Conclusions

With a follow-up of 6 months after RT, LS decrease was predominantly in the endocardial myocardial layer and appeared to be localized in the most exposed part of the left ventricle. For precise evaluation of RT-induced cardiotoxicity and early left ventricular dysfunction, the endocardial layer-based LS might be the most sensitive parameter, in particular to evaluate the impact of radiation exposure during BC RT to the LAD. However, this exploratory analysis remains to be confirmed with larger studies and longer follow-up.

## Data Availability

The datasets used and/or analysed during the current study are available from the corresponding author on reasonable request.

## Abbreviations

3D-CRT: Three-Dimensional Conformal Radiation Therapy
Cx: Circumflex artery
LAD: Left Anterior Descending coronary artery
LS: Longitudinal Strain
LVEF: Left Ventricular Ejection Fraction
RCA: Right Coronary Artery
RT: Radiotherapy (or Radiation Therapy)
